# Association of the *MDM2* SNP285 and SNP309 Genetic Variants with the Risk, Age at Onset and Prognosis of Breast Cancer in Central European Women: A Hospital-Based Case-Control Study

**DOI:** 10.3390/ijms20030509

**Published:** 2019-01-25

**Authors:** Heidi Miedl, Jürgen Lebhard, Lisa Ehart, Martin Schreiber

**Affiliations:** Department of Obstetrics & Gynecology and Comprehensive Cancer Center, Medical University of Vienna, 1090 Vienna, Austria

**Keywords:** breast cancer, *MDM2*, SNP309 (rs2279744), SNP285 (rs117039649), breast cancer risk, age at onset, *TP53* mutation, survival, prognosis

## Abstract

SNP309T>G (rs2279744) and SNP285G>C (rs117039649) in the *MDM2* promoter are thought to have opposite effects on the binding of transcription factor SP1 (specificity protein 1), and consequently on *MDM2* expression, p53 levels, cancer risk, age at onset, and prognosis. Here, we genotyped SNP309 and SNP285 in 406 Austrian breast cancer patients and 254 female controls. The SNP309GG genotype was associated with an increased breast cancer risk in p53 negative (OR, 1.82; 95% CI, 1.09–3.03; *p* = 0.02), but not p53 positive or unselected patients. In contrast, the SNP309TT genotype was associated with an earlier age at onset (TT, 57.0 ± 12.9; TG, 58.6 ± 13.9; GG, 59.7 ± 15.0 years; *p* = 0.048). 31% of SNP309TT, 26% of TG, and 13% of GG tumors were p53 positive (*p* = 0.034), indicating a lower selective pressure to mutate *TP53* in the presence of the G-allele. Moreover, SNP309TT patients exhibited a shortened metastasis-free survival in multivariable analysis. Censoring carriers of the SNP285C-allele hardly altered the strength of these associations of SNP309, thus challenging the proposed antagonistic function of SNP285C towards SNP309G. The minor SNP285C-allele tended to be non-significantly associated with an increased breast cancer risk and a poor disease-free and metastasis-free survival, which may be bystander effects of its complete linkage disequilibrium with SNP309G. We conclude that the SNP309G-allele attenuates the p53-response and leads to a higher breast cancer risk, but also to a later onset of breast cancer and a trend towards a good prognosis.

## 1. Introduction

The tumor suppressor gene *TP53* is the most commonly mutated gene in human cancer [1]. In breast cancer, mutation of *TP53* occurs at a frequency of 25–35% and is often associated with an earlier age at onset, a poor prognosis and a triple-negative molecular subtype [2,3,4,5]. p53 is a transcription factor and maintains genomic integrity primarily by regulating downstream target genes to induce apoptosis, cell cycle arrest, senescence, or other stress responses to halt the progression of cancer [6,7]. In addition to mutation, decreasing the level of wildtype *TP53* is also a major mechanism of functional inactivation of p53 [8]. MDM2 (Mouse Double Minute 2 homolog) is a key negative regulator of p53, being both a target gene of p53 and an E3 ubiquitin ligase that targets p53 for proteasomal degradation, which maintains low p53 protein levels in the absence of stress [8,9,10]. *Mdm2* knockout in mice leads to early embryonic lethality, whereas mice with a deletion of both *Mdm2* and *p53* are viable and develop normally, demonstrating that the negative regulation of p53 is a critical role of MDM2 [11,12]. Accordingly, *MDM2* amplification and/or overexpression may be an important mechanism of aberrant p53 inactivation, and has been observed in many tumors, often mutually exclusive with *TP53* mutation [10,13].

SNP309T>G (rs2279744) is a sequence variant in the *MDM2* intronic promoter P2, and its minor G-allele was found to increase binding of transcription factor SP1, P2 promoter activity, and *MDM2* expression [14]. In the initial study, the SNP309G-allele was found associated with a younger age at onset among individuals with Li-Fraumeni syndrome [14]. However, most subsequent studies of various types of sporadic cancer did not find such a premature onset associated with the G-allele, and revealed inconsistent results with respect to cancer risk. Overall, most studies in Asian populations showed an association of the SNP309GG genotype with an increased cancer risk, whereas studies in Caucasians predominantly found no association [15,16,17,18,19].

A potential mechanistic explanation for these discordant results was provided by the more recent discovery of another SNP in the *MDM2* P2 promoter, SNP285G>C (rs117039649) [20,21]. SNP285 is only 24 bp upstream of SNP309 and in complete linkage disequilibrium with it. The minor SNP285C allele was found exclusively in Caucasians, and population genetics has revealed that it has arisen on a pre-existing SNP309G allele after the separation between Caucasians and modern day East Asians [22]. Like SNP309, SNP285 is also located at the edge of a (different) SP1 binding site, however, whereas the SNP309G allele enhances binding of SP1, the SNP285C allele reduces binding, and might thus antagonize the molecular effects of SNP309G [20]. In addition, SNP285 is located within an estrogen receptor binding site (ERE) that overlaps with the SP1 site [23]. Only a few studies, mostly in Northwestern European populations, have analyzed the association of SNP285 with cancer risk, and revealed a trend for SNP285C to be associated with a reduced risk of gynecological cancers, but not other cancer types [20,21,23,24,25,26,27,28,29]. Some of these studies have found a decreased breast cancer risk associated with the SNP285C allele among carriers of the SNP309GG genotype, but not among carriers of the SNP309TG genotype, and not among subjects unselected for the SNP309 genotype [20,21,24,25,29,30].

The aim of our study was to determine the distribution and allele frequencies of *MDM2* SNP309 and SNP285 in a Central European (Austrian) study population. This closes an important geographical gap between the many Northern and several Southern European populations studied thus far, particularly in light of their divergent SNP285 allele frequencies. We further sought to analyze the association of SNP285 and SNP309 with breast cancer risk, with the age at breast cancer onset, with key clinical and histopathological parameters of breast cancer, in particular the p53 status, and with breast cancer survival and prognosis. By studying the potential mutual interference between SNP309 and SNP285 with respect to these associations, we aimed to address the hypothesis that SNP285 antagonizes the effects of SNP309, thus potentially confounding the many studies of SNP309 in Caucasians that have not taken SNP285 into account.

## 2. Results

### 2.1. Distribution of MDM2 SNP285 and SNP309 Genotypes

The promoter SNPs rs117039649 (SNP285G>C) and rs2279744 (SNP309T>G) in intron 1 of the *MDM2* gene (hereafter referred to as SNP285 and SNP309, respectively) were genotyped in an Austrian hospital-based case-control study of 406 breast cancer patients and 254 control subjects. Tables S1 and S2 show the clinical and histopathological characteristics as well as the frequency of the SNP285 and SNP309 genotypes within the study population. The control population was in Hardy-Weinberg equilibrium for both SNP285 (*p* = 0.34) and SNP309 (*p* = 0.39). Likewise, the patient population was in Hardy-Weinberg equilibrium for both SNPs (*p* = 0.76 for SNP285; *p* = 0.32 for SNP309). The frequency of the minor G-allele of SNP309 (MAF) was 0.365 in patients and 0.347 in controls, close to the 0.367 MAF reported by the 1000 Genomes Project population database [31]. SNP285 genotype GC was observed in 12/254 controls (4.7%; MAF = 0.024), and 23/406 patients (5.7%; MAF = 0.028), all others exhibited genotype GG (Table S1). This frequency of the GC genotype in Austria is somewhat lower than the >7% frequency observed in Northwestern European populations from Norway, UK, and the Netherlands, and closer to the frequencies reported for Southern and Eastern European populations [20,22,25,31]. The SNP285C-allele was observed only in carriers of the SNP309G-allele (genotype GG or TG), consistent with previous reports [20,22,25]. Accordingly, SNP285 and SNP309 were in complete linkage disequilibrium in our study population (D′ = 0.998; *p* = 1.1 × 10^−15^), indicating that the C-allele of SNP285 is present exclusively in a distinct SNP285C/SNP309G haplotype. 9.6% (17/178) of SNP309TG patients (8.2% of controls), and 10.2% (6/59) of SNP309GG patients (7.4% of controls) exhibited the SNP285GC genotype. 

### 2.2. MDM2 SNP309, SNP285, and Breast Cancer Risk

To assess the breast cancer risk associated with the different SNP309 genotypes and alleles, odds ratios (OR), 95% confidence intervals (CI), and *p*-values were determined. This analysis revealed moderately, but non-significantly increased odds ratios associated with the GG genotype, but not the TG genotype, indicating a recessive inheritance mode (Table 1). Censoring individuals with the SNP285GC genotype, or adjusting for SNP285, did not appreciably alter this association, consistent with previous studies [20,25,32] (Table 1). 

We next analyzed the association of SNP285 with breast cancer risk. SNP285CC patients or controls were not observed in our study population, so we compared genotype GC vs. GG, which revealed no significant association (Table 2). Excluding individuals with the SNP309TT genotype from the analysis (which exclusively exhibited the SNP285GG genotype), or adjusting for SNP309, did not appreciably affect the observed odds ratios. Moreover, no significant association of SNP285 with breast cancer risk was observed in SNP309TG or SNP309GG individuals (Table 2).

Since associations of SNP309 with the age at onset, estrogen receptor (ER) status and p53 status have been reported [14,17,33,34,35], odds ratios in relevant breast cancer subpopulations were determined. In this exploratory analysis, no significant associations of SNP309 with breast cancer risk were observed in any subpopulation under a dominant inheritance model (Table S3. In contrast, a significantly elevated odds ratio associated with the GG genotype was observed in p53 negative patients in a recessive inheritance model (OR, 1.82; 95% CI, 1.09–3.03; *p* = 0.02; Table 3). A significant association was also observed in patients with >10% Ki67-positive tumor cells (OR, 1.90; 95% CI, 1.04–3.47; *p* = 0.04). A trend at borderline significance for an elevated odds ratio in the recessive model was also observed in patients older than 55 years, in patients with large tumors (pT2-4; >2 cm), and in stage II–IV patients (Table 3). Analyses of SNP285GC vs. GG did not reveal any association in these subgroups (Table 3). The associations of SNP285GC vs. GG predominantly exhibited the same pattern as that of SNP309GG vs. TT + TG. Accordingly, higher odds ratios for both SNPs were observed in p53 negative than in p53 positive patients, as well as in ER negative, PR positive, Ki67 high, high stage, low grade, and pT2-4 patients, which may be a consequence of their complete linkage disequilibrium (Table 3).

### 2.3. Association of MDM2 SNP309 with Age at Breast Cancer Onset

**Figure 1:**
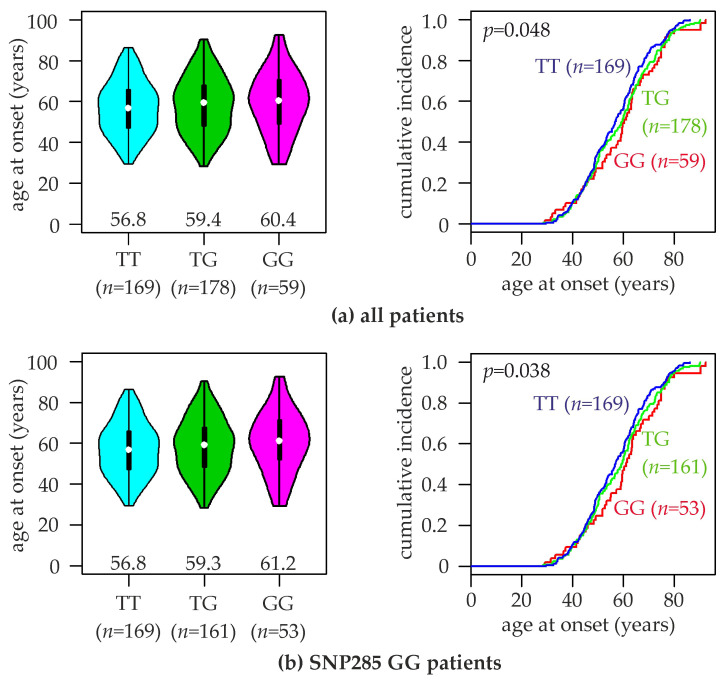
Association of SNP309 genotypes with the age at breast cancer onset. Violin plots (left) and curves of the cumulative breast cancer incidence (right) of the indicated age at onset are shown for (**a**) all patients of the study population, and (**b**) patients with the SNP285GG genotype. SNP309 genotypes (TT, TG, GG) and numbers of patients (*n*) are indicated. Numbers in left panels indicate the median age at breast cancer onset of each genotype (indicated by white dots).

Associations of SNP309 with the onset age of hereditary breast cancer have been reported in several studies [14,17,34,36,37,38,39,40]. In our study population, we found the mean age of breast cancer onset for patients with the TT, TG, and GG genotype to be 57.0 ± 12.9 years (median, 56.8), 58.6 ± 13.9 years (median, 59.4), and 59.7 ± 15.0 years (median, 60.4), respectively (*p* < 0.05, log-rank test; Figure 1a). When carriers of the SNP285GC genotype were excluded from this analysis, somewhat more pronounced differences in the age at onset were observed: TT, 57.0 ± 12.9 years (median, 56.8; unaltered since none of them was SNP285GC); TG, 58.5 ± 13.7 years (median, 59.3); GG, 60.4 ± 14.9 years (median, 61.2; *p* < 0.04; Figure 1b). A similar trend to an approximately 3–4 years younger mean and median age at onset associated with the TT genotype was also observed in ER positive patients, but not in p53 negative patients (Figure S1. No significant differences in the age at onset were observed between patients with the SNP285GG vs. GC genotypes (Figure S1).

### 2.4. Association of MDM2 SNP309 Genotype with p53 Status

The p53 status was retrieved from clinical records, which had been determined by immune histochemistry (IHC) as part of the routine clinical breast cancer diagnostics in Austria. p53 IHC positivity indicates an inactivating mutation of p53, since it leads to a defective p53-MDM2 negative feedback loop and hence an accumulation of stabilized p53 protein [5,41,42,43,44,45]. The presence of ≥10% p53-positive tumor cell nuclei was scored as p53 positive. p53 status was then analyzed as a function of SNP309 genotype (Table 4). The fraction of p53-positive tumors was 31.1% (47/151) in SNP309TT patients, 25.9% (38/147) in TG patients, but only 13.2% (7/53) in GG patients (*p* = 0.03; Fisher’s exact test), indicating a higher rate of p53 mutation with increasing number of SNP309 T-alleles (Table 4). The rate of high p53 positivity (>60% p53 positive tumor cells; clinical score 3) also followed this trend, being 17.2% (26/151) in TT patients, 12.9% (19/147) in TG patients, and 5.7% (3/53) in GG patients (Table 4). These data may reflect a higher selective pressure to mutate *TP53* if the SNP309 T-allele is present.

### 2.5. Association of MDM2 SNP309 Genotype with Breast Cancer Prognosis

**Figure 2:**
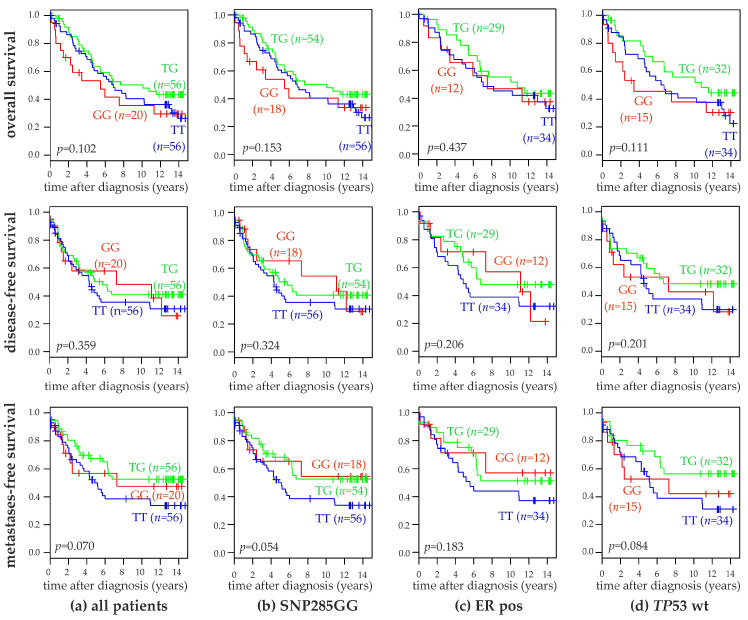
Association of SNP309 genotypes with the survival of human breast cancer patients. Kaplan-Meier analyses of the overall survival (top panels), disease-free survival (middle panels), and metastasis-free survival (bottom panels) in unselected patients (**a**; *n* = 132), patients with the SNP285 GG-genotype (**b**; *n* = 128), ER positive patients (**c**; *n* = 75) and *TP53* wildtype patients (**d**; *n* = 81) are shown. Patient subgroups with SNP309 genotypes TT, TG, and GG as well as their numbers (*n*) are indicated in each panel. ER, estrogen receptor; and wt, wildtype.

The overall survival (OS), disease-free survival (DFS) and metastasis-free survival (MFS) of patients with the three SNP309 genotypes were compared in Kaplan-Meier analyses of unselected patients, SNP285GG patients, ER-positive patients, and patients with wildtype *TP53*. Detailed follow-up records for this survival analyses were available for 132 of the genotyped patients. For 115 of these 132 patients, *TP53* status was determined by DNA sequencing in addition to IHC [46], and these sequencing data were used in the survival analyses. No significant differences in any of these analyses were observed, although the TT-genotype tended to be associated with a slightly poorer metastasis-free survival (Figure 2). Accordingly, multivariable Cox proportional hazards regression analyses of the disease-free and metastasis-free survival of the variables SNP309 genotype, SNP285 genotype, ER-status, p53-status, and lymph node status were performed. Each variable was analyzed individually in parallel (Table 5 and Table 6). Lymph node status, i.e., the presence vs. absence of lymph node metastases was the strongest prognostic marker in these analyses. ER-status considerably gained prognostic power in the multivariable compared to the univariable analyses, whereas p53-status showed the opposite behavior. The SNP309T-allele was significantly associated with a poorer survival in the multivariable analysis of the metastasis-free survival (*p* < 0.04; Table 6). Conversely, the SNP285GC-genotype was non-significantly associated with a poorer survival in both analyses (Table 5 and Table 6).

## 3. Discussion

The discovery of SNP285 in *MDM2* promoter P2 has led to an elegant hypothesis potentially solving a long-standing conundrum: Why is the SNP309G-allele associated with an increased cancer risk in Asian, but not in Caucasian populations [16,17,18,19,20,21,27,29]? According to this hypothesis, SNP309 likely is associated with cancer risk, consistent with the finding that genetically engineered SNP309GG mice are more tumor-prone than SNP309TT mice [47]. However, this association is apparently confounded in Europeans by the adjacent SNP285, which also affects SP1 binding to another site, antagonizes the effects of SNP309, and is present at a low frequency in Europeans, but absent in Asians [20,22]. On the other hand, the present study, together with previous ones, found no evidence to support this hypothesis. Like many previous studies in European populations, we found that SNP309 is not significantly associated with breast cancer risk, although a trend for an increased risk associated with the GG genotype in the recessive inheritance model was observed [16,18,19]. However, when we corrected for the potential confounding effect of SNP285, we still did not observe any significant associations. In fact, the observed odds ratios associated with SNP309 genotypes hardly changed upon exclusion of carriers of the SNP285C allele or adjustment for SNP285, consistent with previous reports [25,32]. We conclude that the discordant results between European and Asian populations with respect to the association of SNP309 with cancer risk must be due to differences other than the presence vs. absence of the variant C-allele of SNP285.

In the initial study of SNP309, the GG genotype was found to be associated with a significantly younger age at cancer onset in Li-Fraumeni patients [14]. This finding was confirmed in most subsequent studies in Li-Fraumeni patients [36,37,38,39,40]. Interestingly, an association of the SNP309GG genotype with an earlier onset was also observed in several studies of familial breast cancer, particularly in *BRCA1* mutation carriers [17,27,48,49]. However, most studies of sporadic breast cancer did not find such a premature onset associated with the SNP309G-allele [18,21,50,51,52,53]. Here we report an effect opposite to that in familial cancer syndromes: In our study population, the SNP309TT genotype is associated with a younger age at breast cancer onset, consistent with a previous report [17]. Like our study, Lum et al. reported an association of the SNP309GG genotype with an increased breast cancer risk, but a delayed onset [17]. This is unexpected given the proposed molecular function of the SNP309G-allele to increase the expression of *MDM2* and hence to attenuate the p53-pathway. We hypothesize that this seemingly paradoxical association may be due to the differential rate of p53 positivity found by us and others, which is low in SNP309GG, but high in TT-tumors (see discussion below). It has been convincingly established that *TP53* mutant patients have a much younger age at onset [2,3,4]. Accordingly, we propose that the SNP309TT genotype leads to a higher selective pressure to mutate *TP53* and hence to a much higher rate of these mutations, which then causes the earlier mean age at onset associated with that genotype. This effect of differential somatic *TP53* mutation rate obviously does not exist in Li-Fraumeni patients who have a germline mutation in *TP53*, which may explain why they differ from sporadic cancer cases with respect to the association of SNP309 with the age at cancer onset. Consistently, we found an association of the age at breast cancer onset with *MDM2* mRNA expression. Significantly elevated mean *MDM2* mRNA levels were observed in patients with an age ≥55 years, as well as in post-menopausal patients (H. Miedl and M. Schreiber, unpublished data).

We found that the SNP309GG genotype is significantly associated with an increased breast cancer risk in p53 negative patients, who harbour a functional p53 protein (OR, 1.82; 95% CI; 1.09–3.03; and *p* = 0.02), but not in p53 positive or unselected patients (Table 1 and Table 3), consistent with a previous report [50]. Thus, the impact of SNP309 on breast cancer risk appears to be limited to tumors in which p53 is functional, which is biologically plausible since the key function of *MDM2* to negatively regulate p53 becomes irrelevant if p53 is inactivated by mutation. Moreover, we found evidence for an inverse correlation of *TP53* mutation with the presence of the variant SNP309G-allele. *TP53* was positive in 31% of SNP309TT patients, 26% of TG patients, but only 13% of GG patients (*p* < 0.04; Table 4). Since the SN309G allele leads to a higher expression of *MDM2* and consequently lower levels of p53, presence of the SNP309TG and particularly the GG genotype may, at least in part, functionally substitute the inactivation of *TP53* by mutation. Thus, if p53 tumor suppressor activity is already attenuated by overexpression of *MDM2*, the selective pressure to additionally mutate *TP53* may be reduced in the presence of the SNP309G allele, and this may be reflected in the lower rates of p53 positivity observed in carriers of the SNP309GG and TG genotypes. Interestingly, amplification of *MDM2*, another clinically relevant mechanism in carcinogenesis to increase *MDM2* and decrease p53 expression, was also found to be associated with a reduced rate of *TP53* mutation [10,13,54]. Like in the present study, a reduced rate of *TP53* mutations in carriers of the SNP309G allele has been reported for non-small cell lung cancer [55], hepatocellular carcinoma [35], and bladder cancer [56]. In contrast, no significant association was found in oral cancer, pancreatic cancer, and two studies of colorectal cancer [57,58,59,60]. A lower frequency of p53 positivity was also found in breast cancer patients with the SNP309GG genotype compared to those with the SNP309TT genotype [50]. An analogous association of a germline polymorphism with a differential mutation rate of *TP53* was also found for the R72P coding SNP (rs1042522) in *TP53* itself [46,61].

We found no significant association of SNP309 genotype with survival, consistent with most reports in breast cancer and other tumor types [50,62,63,64,65]. However, the TT genotype tended to be associated with a slightly poorer metastasis-free survival, and this association was significant in multivariable analysis (Table 6). Since the SNP309T allele is thought to lead to lower MDM2 and hence higher p53 levels [14], we expected it to rather be associated with an improved survival. However, the TT genotype has been found associated with a poor prognosis previously, including in a breast cancer study in BRCA1/2 mutation carriers [66,67,68]. We hypothesize that this counter-intuitive association is due to the much higher rate of *TP53* mutation in SNP309TT patients. To the best of our knowledge, we here report the first analysis of the association of SNP285 with breast cancer survival. We found the SNP285GC genotype non-significantly associated with a poorer disease-free and metastasis-free survival in univariable and multivariable analyses (Table 5 and Table 6). However, this result is based on the analysis of only four patients with the GC genotype and 128 with the SNP285GG genotype and should be interpreted with caution. The association of SNP285 with survival has previously been analyzed in two lung cancer studies. One study found no association, whereas the other study found an association of the SNP285GC genotype with a worse progression-free survival compared to the GG genotype, but no association with the overall survival [32,55].

## 4. Patients and Methods

### 4.1. Study Population

This study was approved and is annually reviewed by the Institutional Review Board (“Ethikkommission”) of the Medical University of Vienna, Austria (MUV) (protocol 141/2002, 31 August 2005). Written informed consent was obtained from all participants. Clinical and histopathological characteristics of the study population are shown in Table S1. 276 consecutive breast cancer patients treated between 2002 and 2004, and another 134 patients treated between 1991 and 1994 at the Department of Obstetrics and Gynecology, MUV, were enrolled in this study. From the latter 134 patients, detailed follow-up records were available. Patients with benign gynecological lesions and healthy females without any malignancies in their personal history were enrolled as controls between 2002 and 2004 at the Department of Obstetrics and Gynecology, MUV (*n* = 255). Only women of Central European descent from the same geographical area were included as patients or controls. The response rates for both patients and controls were >80%. Two patients were excluded from further analyses due to failure to genotype SNP309, one additional patient and one control due to failure to genotype SNP285, and one additional patient since genotyping of both SNPs was unsuccessful. Accordingly, all analyses reported here are based on the 406 breast cancer patients and 254 controls for whom both SNP309 and SNP285 were successfully genotyped.

### 4.2. DNA Isolation and Genotyping

Genomic DNA has been extracted previously from peripheral lymphocytes with the QIAamp DNA Blood Midi kit (Qiagen, Venlo, The Netherlands), and from fresh-frozen primary tumour samples with the High Pure PCR Template Preparation Kit (Roche, Vienna, Austria) as described [46,69]. Genotyping of SNP rs117039649 (SNP285; c.-315G>C; g.5586G>C) and SNP rs2279744 (SNP309; c.-291T>G; g.5610T>G) in intron 1 of the *MDM2* gene was performed by qPCR with TaqMan Genotyping Mastermix and the following primers and allele-specific, fluorescently labeled probes (Applied Biosystems, Brunn/Gebirge, Austria): SNP285, forward primer, 5′-CGCGGGAGTTCAGGGTAAAG-3′; reverse primer: 5′-ACAGGCACCTGCGATCATC-3′; G-allele reporter probe: 5′-CCGGCCCCCGTGAC-3′; C-allele reporter probe: 5′-CGGCCCGCGTGAC-3′; SNP309, forward primer, 5′-CGGGAGTTCAGGGTAAAGGT-3′; reverse primer: 5′-ACAGGCACCTGCGATCATC-3′; T-allele reporter probe: 5′-CCGCGCCGAAGCG-3′; G-allele reporter probe: 5′-CCGCGCCGCAGCG-3′. PCR reactions were carried out on a CFX96 instrument for SNP285 (BioRad, Vienna, Austria), and a QS3 instrument for SNP309 (Applied Biosystems, Brunn/Gebirge, Austria) with 20 ng of genomic DNA in a reaction volume of 20 µL following the manufacturer’s instructions. As a quality control, ~100 samples were genotyped in duplicate, 5 of them from separate DNA isolations, with 100% concordance. Laboratory staff was blinded with respect to the identity of these duplicates, and with respect to patient or control status of all samples. For technical reasons, the SNP285 genotype could not be determined for two patients and one control subject, and the SNP309 genotype for three patients. 

### 4.3. Statistical Analyses

Statistical analyses were performed with R 3.3.2, an open-source language and environment for statistical computing [70]. Chi-square tests with Yates’ continuity correction were used to evaluate whether the study population was in Hardy-Weinberg equilibrium. Confidence intervals and *p*-values associated with odds ratios were calculated by the mid-P exact method. As advocated [71], we did not adjust our subgroup analyses (Table 3) for multiple testing, since they should be considered exploratory. Differences between two groups with respect to age at onset were analyzed by two-sided *t*-tests, and between three or more groups by ANOVA. Statistical methods of survival analyses, as well as follow-up details of our study population have been described in References [46,72]. *p*-Values to Kaplan-Meier analyses and analyses of the cumulative breast cancer incidence were calculated by log-rank tests, as described in Reference [73]. The disease-free and metastasis-free survival were also analyzed by using a Cox proportional hazard model, unadjusted or adjusted for ER status, *TP53* status, and lymph node status. The subcategories of these variables were coded as follows: SNP309 genotype, TT = 0, TG = 1, GG = 2; SNP285 genotype, GG = 0, GC = 1; ER status, positive = 0, negative = 1; *TP53* status, wildtype = 0, mutated = 1 (determined by sequencing [46]); lymph node status, absence of lymph node metastases (pN0) = 0, presence of lymph node metastases (pN+) = 1. All *p*-values shown are two-sided. Associations with *p*-values < 0.05 were considered statistically significant.

## 5. Conclusions

We found that the SNP309GG genotype was associated with an older age at breast cancer onset, a good prognosis in multivariable analysis of the metastasis-free survival and a trend in the same direction in related survival analyses, an increased breast cancer risk in specific subgroups, and a rate of p53 positivity less than half compared to the SNP309TT genotype. These findings are consistent with a model in which the p53-response is attenuated in the presence of the SNP309GG genotype. A direct consequence of this attenuation may be that the risk of breast cancer and other cancers is increased, but that the selective pressure to mutate *TP53* is reduced. An attenuated p53-response is presumably still functional to some extent as opposed to one that is completely inactivated by mutation, and consequently leads to a later onset of breast cancer and a trend towards a good prognosis. None of the observed associations of SNP285 were significant in our study, which may in part be due to the very low frequency of the variant C-allele. However, the observed trends towards an increased breast cancer risk and a poor survival are at odds with the proposed molecular function of the SNP285C-allele to reduce binding of SP1 and expression of *MDM2*. Accordingly, we conclude that these associations of SNP285C are bystander effects of its complete linkage disequilibrium with SNP309G. The odds ratios in subgroups according to SNP309 genotype (Table 2), and in clinical subgroups (Table 3) support this conclusion. For example, the odds ratios associated with both the SNP309GG and the SNP285GC genotype are higher in the Ki67-high than in the Ki67-low subgroup (Table 3). This is consistent with the molecular function of SNP309G to attenuate the anti-proliferative p53-response, but not with that of SNP285C to enhance it. These bystander effects may be more pronounced in our study population compared to previous ones due to the lower frequency of the SNP285GC genotype and the complete absence of SNP285CC subjects. Finally, the fact that SNP309 is associated with an increased cancer risk in most Asian but not European studies has been suggested to be due to the antagonistic molecular function and consequential confounding effect of SNP285, which is present in Europeans, but absent in Asians. However, the results of the present study do not support this hypothesis. Specifically, when we excluded SNP285GC individuals from our analyses, thus mimicking the situation in Asian populations, the observed associations (ORs) were still not significant, and remained essentially unaltered (Table 1). In conclusion, we report here the first analysis of SNP285 in a Central European study population, the first analysis of the association of SNP285 with breast cancer survival, the first analysis of SNP285 in clinically relevant subgroups of breast cancer, and the first analysis of potential confounding effects of SNP285 on the association of SNP309 with the age at onset and survival.

## Figures and Tables

**Table 1:** Association of *MDM2* SNP309 genotypes and alleles with breast cancer risk.

Genotypes/Alleles	All subjects			SNP285GG Subjects			All Subjects		
	Unadjusted			Unadjusted			Adjusted for SNP285		
	OR	95% CI	*p*-Value	OR	95% CI	*p*-Value	OR	95% CI	*p*-Value
**GG vs. TT**	1.36	0.81–2.28	0.278	1.32	0.77–2.25	0.321	1.33	0.79–2.24	0.308
**GG vs. TG**	1.50	0.90–2.52	0.117	1.47	0.87–2.54	0.169	1.49	0.90–2.50	0.116
**GG vs. TG + TT**	1.43	0.88–2.32	0.143	1.39	0.84–2.31	0.191	1.42	0.87–2.31	0.152
**TG vs. TT**	0.91	0.65–1.27	0.579	0.89	0.63–1.26	0.513	0.89	0.63–1.25	0.518
**TG + GG vs. TT**	0.99	0.72–1.36	0.942	0.97	0.70–1.34	0.856	0.97	0.70–1.34	0.856
**G vs. T**	1.08	0.86–1.36	0.507	1.06	0.84–1.35	0.620	1.07	0.85–1.35	0.563

Analyses of breast cancer cases vs. controls of the indicated SNP309 genotypes or alleles are shown. Analyses were performed in all subjects or subjects with the SNP285 genotype GG, unadjusted or adjusted for SNP285 genotype as indicated. OR, odds ratios; 95% CI, 95% confidence intervals.

**Table 2:** Association of *MDM2* SNP285 genotypes with breast cancer risk.

In Subgroup by SNP309 Genotype	SNP285 Genotype (*n*)				SNP285 GC vs. GG		
	Controls		Patients				
	GG	GC	GG	GC	OR	95% CI	*p*-Value
**All Genotypes**	242	12	383	23	1.21	0.59–2.48	0.597
**TG and GG**	137	12	214	23	1.23	0.59–2.55	0.580
**TG**	112	10	161	17	1.18	0.52–2.68	0.686
**GG**	25	2	53	6	1.42	0.27–7.51	0.677
**Adjusted SNP309**	242	12	383	23	1.23	0.59–2.55	0.583

Analyses of SNP285 GC vs. GG genotypes in breast cancer cases vs. controls in subpopulations with the indicated SNP309 genotypes are shown. OR, odds ratios; and 95% CI, 95% confidence intervals.

**Table 3:** Association of *MDM2* SNP309 and SNP285 with breast cancer risk in patient subpopulations.

	Patients			SNP309			SNP285		
	Subgr.	Number (%)	OR		95% CI	*p*	OR	95% CI	*p*
Age (years) ^1^	<55	171 (42.1%)	1.18		0.64–2.16	0.599	1.39	0.60–3.22	0.449
	≥55	235 (57.9%)	1.62		0.96–2.75	0.071	1.09	0.48–2.47	0.845
Menopausal status	pre	100 (28.6%)	1.37		0.69–2.73	0.380	1.52	0.58–3.97	0.404
	post	250 (71.4%)	1.55		0.93–2.63	0.097	0.93	0.40–2.14	0.861
p53 status	pos	92 (26.2%)	0.69		0.29–1.65	0.393	0.68	0.19–2.47	0.543
	neg	259 (73.8%)	1.82		1.09–3.03	0.020	1.51	0.71–3.19	0.281
ER status	pos	265 (68.1%)	1.36		0.80–2.32	0.247	1.21	0.56–2.64	0.631
	neg	124 (31.9%)	1.62		0.87–3.01	0.135	1.39	0.55–3.50	0.488
PR status	pos	185 (48.3%)	1.50		0.85–2.64	0.162	1.40	0.61–3.19	0.425
	neg	198 (51.7%)	1.44		0.82–2.53	0.200	1.19	0.51–2.75	0.691
HER2 status	pos	74 (20.0%)	1.47		0.69–3.12	0.329	1.15	0.36–3.68	0.813
	neg	296 (80.0%)	1.39		0.83–2.33	0.207	1.31	0.62–2.77	0.483
Ki67 status	≤10%	183 (59.4%)	1.46		0.82–2.53	0.199	0.80	0.31–2.08	0.647
	>10%	125 (40.6%)	1.90		1.04–3.47	0.040	2.14	0.93–4.91	0.075
Stage	0 or I	143 (43.5%)	1.06		0.55–2.04	0.864	0.88	0.32–2.41	0.807
	II–IV	186 (56.5%)	1.62		0.92–2.83	0.092	1.27	0.55–2.94	0.581
Grade	pG1–2	237 (61.9%)	1.56		0.91–2.65	0.101	1.36	0.62–2.97	0.436
	pG3	146 (38.1%)	1.33		0.72–2.48	0.363	1.17	0.47–2.93	0.740
Tumor Size	pT1	175 (52.6%)	1.15		0.63–2.10	0.659	1.09	0.45–2.65	0.844
	pT2–4	158 (47.4%)	1.66		0.93–2.96	0.089	1.36	0.57–3.23	0.485

SNP309, GG vs. TT + TG (recessive model); SNP285, GC vs. GG; Subgr., subgroup of patients; OR, odds ratios; 95% CI, 95% confidence intervals; *p*, *p*-values; pre, pre-menopausal; post, post-menopausal; ER, estrogen receptor; PR, progesterone receptor; pos, positive; and neg, negative; ^1^ patients aged under 55 years or ≥55 years at diagnosis were compared to control subjects of any age.

**Table 4:** Association of *MDM2* SNP309 genotypes with p53 status.

p53 Status	Total	TT	TG	GG	*p*
neg (0)	259 (74%)	104 (69%)	109 (74%)	46 (87%)	0.034
pos (1–3)	92 (26%)	47 (31%)	38 (26%)	7 (13%)	
score 1	24 (7%)	12 (8%)	10 (7%)	2 (4%)	0.948
score 2	20 (6%)	9 (6%)	9 (6%)	2 (4%)	
score 3	48 (14%)	26 (17%)	19 (13%)	3 (6%)	

Numbers of patients in the indicated subgroups are shown. Numbers in parentheses show the fraction of patients (%) with the indicated p53 status. Number of p53-positive tumor cells: p53 negative (neg), <10%; score 0; score 1, 10–30%; score 2, 30–60%; and score 3, >60%. Score 1–3 is considered as p53 positive (pos). *p*, *p*-value, determined by Fisher’s exact test.

**Table 5:** Univariable and multivariable analyses of the disease-free survival using a Cox proportional hazards model.

Variable	Univariable			Multivariable		
	HR	95% CI	*p*-Value	HR	95% CI	*p*-Value
**SNP309 Genotype**	0.91	0.65–1.28	0.5820	0.78	0.52–1.16	0.2205
**SNP285 Genotype**	1.88	0.59–5.98	0.2860	1.92	0.58–6.35	0.2829
**ER Status**	1.47	0.94–2.29	0.0877	1.97	1.16–3.34	0.0122
**p53 Status**	1.63	0.98–2.71	0.0613	1.29	0.75–2.22	0.3500
**Lymph Node Status**	2.38	1.45–3.89	0.0006	2.65	1.52–4.62	0.0006

HR, hazard ratio; 95% CI, 95% confidence intervals; ER, estrogen receptor. Subcategories of the indicated variables were coded as described in Methods.

**Table 6:** Univariable and multivariable analyses of the metastasis-free survival using a Cox proportional hazards model.

Variable	Univariable			Multivariable		
	HR	95% CI	*p*-Value	HR	95% CI	*p*-Value
**SNP309 genotype**	0.79	0.54–1.16	0.2220	0.62	0.39–0.97	0.0378
**SNP285 genotype**	2.15	0.67–6.87	0.1980	2.70	0.79–9.25	0.1151
**ER status**	1.35	0.82–2.20	0.2360	1.77	1.00–3.15	0.0506
**p53 status**	1.26	0.72–2.21	0.4200	1.03	0.57–1.86	0.9256
**Lymph node status**	2.98	1.66–5.35	0.0002	3.67	1.93–7.00	0.0001

HR, hazard ratio; 95% CI, 95% confidence intervals; ER, estrogen receptor. Subcategories of the indicated variables were coded, as described in Methods.

## Supplementary Materials

Supplementary materials can be found at http://www.mdpi.com/1422-0067/20/3/509/s1.

## Abbreviations

BRCA1/2	breast cancer gene 1 and 2
CI	confidence interval
DFS	disease-free survival
HR	hazard ratio
ER	estrogen receptor
IHC	immune histochemistry
MAF	minor allele frequency
MDM2	Mouse Double Minute 2 homolog
MFS	metastasis-free survival
neg	negative
OR	odds ratio
OS	overall survival
*p*	*p*-value
pos	positive
PR	progesterone receptor
SNP	single-nucleotide polymorphism
SP1	specificity protein 1
TP53	tumor protein p53

## References

[1] Martincorena I., Campbell P.J. (2015). Somatic mutation in cancer and normal cells. Science.

[2] Bouaoun L., Sonkin D., Ardin M., Hollstein M., Byrnes G., Zavadil J., Olivier M. (2016). TP53 Variations in Human Cancers: New Lessons from the IARC TP53 Database and Genomics Data. Hum. Mutat..

[3] Blandino G., Deppert W., Hainaut P., Levine A., Lozano G., Olivier M., Rotter V., Wiman K., Oren M. (2012). Mutant p53 protein, master regulator of human malignancies: A report on the Fifth Mutant p53 Workshop. Cell Death Differ..

[4] Petitjean A., Achatz M.I., Borresen-Dale A.L., Hainaut P., Olivier M. (2007). TP53 mutations in human cancers: Functional selection and impact on cancer prognosis and outcomes. Oncogene.

[5] Lacroix M., Toillon R.A., Leclercq G. (2006). p53 and breast cancer, an update. Endocr. Relat. Cancer.

[6] Bieging K.T., Mello S.S., Attardi L.D. (2014). Unravelling mechanisms of p53-mediated tumour suppression. Nat. Rev. Cancer.

[7] Kruiswijk F., Labuschagne C.F., Vousden K.H. (2015). p53 in survival, death and metabolic health: A lifeguard with a licence to kill. Nat. Rev. Mol. Cell Biol..

[8] Pant V., Lozano G. (2014). Limiting the power of p53 through the ubiquitin proteasome pathway. Genes Dev..

[9] Wade M., Li Y.C., Wahl G.M. (2013). MDM2, MDMX and p53 in oncogenesis and cancer therapy. Nat. Rev. Cancer.

[10] Burgess A., Chia K.M., Haupt S., Thomas D., Haupt Y., Lim E. (2016). Clinical Overview of MDM2/X-Targeted Therapies. Front. Oncol..

[11] Jones S.N., Roe A.E., Donehower L.A., Bradley A. (1995). Rescue of embryonic lethality in Mdm2-deficient mice by absence of p53. Nature.

[12] Montes de Oca Luna R., Wagner D.S., Lozano G. (1995). Rescue of early embryonic lethality in mdm2-deficient mice by deletion of p53. Nature.

[13] Oliner J.D., Pietenpol J.A., Thiagalingam S., Gyuris J., Kinzler K.W., Vogelstein B. (1993). Oncoprotein MDM2 conceals the activation domain of tumour suppressor p53. Nature.

[14] Bond G.L., Hu W., Bond E.E., Robins H., Lutzker S.G., Arva N.C., Bargonetti J., Bartel F., Taubert H., Wuerl P. (2004). A single nucleotide polymorphism in the MDM2 promoter attenuates the p53 tumor suppressor pathway and accelerates tumor formation in humans. Cell.

[15] Hu Z., Jin G., Wang L., Chen F., Wang X., Shen H. (2007). MDM2 promoter polymorphism SNP309 contributes to tumor susceptibility: Evidence from 21 case-control studies. Cancer Epidemiol. Biomark. Prev..

[16] Economopoulos K.P., Sergentanis T.N. (2010). Differential effects of MDM2 SNP309 polymorphism on breast cancer risk along with race: A meta-analysis. Breast Cancer Res. Treat..

[17] Lum S.S., Chua H.W., Li H., Li W.F., Rao N., Wei J., Shao Z., Sabapathy K. (2008). MDM2 SNP309 G allele increases risk but the T allele is associated with earlier onset age of sporadic breast cancers in the Chinese population. Carcinogenesis.

[18] Schmidt M.K., Reincke S., Broeks A., Braaf L.M., Hogervorst F.B., Tollenaar R.A., Johnson N., Fletcher O., Peto J., Tommiska J. (2007). Do MDM2 SNP309 and TP53 R72P interact in breast cancer susceptibility? A large pooled series from the breast cancer association consortium. Cancer Res..

[19] Zhao E., Cui D., Yuan L., Lu W. (2012). MDM2 SNP309 polymorphism and breast cancer risk: A meta-analysis. Mol. Biol. Rep..

[20] Knappskog S., Bjornslett M., Myklebust L.M., Huijts P.E., Vreeswijk M.P., Edvardsen H., Guo Y., Zhang X., Yang M., Ylisaukko-Oja S.K. (2011). The MDM2 promoter SNP285C/309G haplotype diminishes Sp1 transcription factor binding and reduces risk for breast and ovarian cancer in Caucasians. Cancer Cell.

[21] Paulin F.E., O’Neill M., McGregor G., Cassidy A., Ashfield A., Ali C.W., Munro A.J., Baker L., Purdie C.A., Lane D.P. (2008). MDM2 SNP309 is associated with high grade node positive breast tumours and is in linkage disequilibrium with a novel MDM2 intron 1 polymorphism. BMC Cancer.

[22] Knappskog S., Gansmo L.B., Dibirova K., Metspalu A., Cybulski C., Peterlongo P., Aaltonen L., Vatten L., Romundstad P., Hveem K. (2014). Population distribution and ancestry of the cancer protective MDM2 SNP285 (rs117039649). Oncotarget.

[23] Knappskog S., Trovik J., Marcickiewicz J., Tingulstad S., Staff A.C., Romundstad P., Hveem K., Vatten L., Salvesen H.B., Lonning P.E. (2012). SNP285C modulates oestrogen receptor/Sp1 binding to the MDM2 promoter and reduces the risk of endometrial but not prostatic cancer. Eur. J. Cancer.

[24] Wang P., Wang M., Li S., Ma L., Xi S., He J. (2016). Association of the MDM2 SNP285 Polymorphism with Cancer Susceptibility: A Meta-Analysis. Dis. Mark..

[25] Gansmo L.B., Knappskog S., Romundstad P., Hveem K., Vatten L., Lonning P.E. (2015). Influence of MDM2 SNP309 and SNP285 status on the risk of cancer in the breast, prostate, lung and colon. Int. J. Cancer.

[26] Vargas-Torres S.L., Portari E.A., Klumb E.M., Guillobel H.C., Camargo M.J., Russomano F.B., Macedo J.M. (2014). Effects of MDM2 promoter polymorphisms on the development of cervical neoplasia in a Southeastern Brazilian population. Biomarkers.

[27] Bjornslett M., Knappskog S., Lonning P.E., Dorum A. (2012). Effect of the MDM2 promoter polymorphisms SNP309T>G and SNP285G>C on the risk of ovarian cancer in BRCA1 mutation carriers. BMC Cancer.

[28] Knappskog S., Lonning P.E. (2011). MDM2 promoter SNP285 and SNP309; phylogeny and impact on cancer risk. Oncotarget.

[29] Zhang J., Zhang Y., Zhang Z. (2018). Association of rs2279744 and rs117039649 promoter polymorphism with the risk of gynecological cancer: A meta-analysis of case-control studies. Medicine (Baltimore).

[30] Piotrowski P., Lianeri M., Rubis B., Knula H., Rybczynska M., Grodecka-Gazdecka S., Jagodzinski P.P. (2012). Murine double minute clone 2309T/G and 285G/C promoter single nucleotide polymorphism as a risk factor for breast cancer: A Polish experience. Int. J. Biol. Mark..

[31] The 1000 Genomes Project Consortium (2015). A global reference for human genetic variation. Nature.

[32] Ryan B.M., Calhoun K.M., Pine S.R., Bowman E.D., Robles A.I., Ambs S., Harris C.C. (2012). MDM2 SNP285 does not antagonize the effect of SNP309 in lung cancer. Int. J. Cancer.

[33] Bond G.L., Levine A.J. (2007). A single nucleotide polymorphism in the p53 pathway interacts with gender, environmental stresses and tumor genetics to influence cancer in humans. Oncogene.

[34] Bond G.L., Hirshfield K.M., Kirchhoff T., Alexe G., Bond E.E., Robins H., Bartel F., Taubert H., Wuerl P., Hait W. (2006). MDM2 SNP309 accelerates tumor formation in a gender-specific and hormone-dependent manner. Cancer Res..

[35] Acun T., Terzioglu-Kara E., Konu O., Ozturk M., Yakicier M.C. (2010). Mdm2 Snp309 G allele displays high frequency and inverse correlation with somatic P53 mutations in hepatocellular carcinoma. Mutat. Res..

[36] Bougeard G., Baert-Desurmont S., Tournier I., Vasseur S., Martin C., Brugieres L., Chompret A., Bressac-de Paillerets B., Stoppa-Lyonnet D., Bonaiti-Pellie C. (2006). Impact of the MDM2 SNP309 and p53 Arg72Pro polymorphism on age of tumour onset in Li-Fraumeni syndrome. J. Med. Genet..

[37] Marcel V., Palmero E.I., Falagan-Lotsch P., Martel-Planche G., Ashton-Prolla P., Olivier M., Brentani R.R., Hainaut P., Achatz M.I. (2009). TP53 PIN3 and MDM2 SNP309 polymorphisms as genetic modifiers in the Li-Fraumeni syndrome: Impact on age at first diagnosis. J. Med. Genet..

[38] Renaux-Petel M., Sesboue R., Baert-Desurmont S., Vasseur S., Fourneaux S., Bessenay E., Frebourg T., Bougeard G. (2014). The MDM2 285G-309G haplotype is associated with an earlier age of tumour onset in patients with Li-Fraumeni syndrome. Fam. Cancer.

[39] Ruijs M.W., Schmidt M.K., Nevanlinna H., Tommiska J., Aittomaki K., Pruntel R., Verhoef S., Van’t Veer L.J. (2007). The single-nucleotide polymorphism 309 in the MDM2 gene contributes to the Li-Fraumeni syndrome and related phenotypes. Eur. J. Hum. Genet..

[40] Wu C.C., Krahe R., Lozano G., Zhang B., Wilson C.D., Jo E.J., Amos C.I., Shete S., Strong L.C. (2011). Joint effects of germ-line TP53 mutation, MDM2 SNP309, and gender on cancer risk in family studies of Li-Fraumeni syndrome. Hum. Genet..

[41] Midgley C.A., Lane D.P. (1997). p53 protein stability in tumour cells is not determined by mutation but is dependent on Mdm2 binding. Oncogene.

[42] Borresen-Dale A.L. (2003). TP53 and breast cancer. Hum. Mutat..

[43] Geisler H.E., Geisler J.P., Miller G.A., Geisler M.J., Wiemann M.C., Zhou Z., Crabtree W. (2001). p21 and p53 in ovarian carcinoma: Their combined staining is more valuable than either alone. Cancer.

[44] Kaserer K., Schmaus J., Bethge U., Migschitz B., Fasching S., Walch A., Herbst F., Teleky B., Wrba F. (2000). Staining patterns of p53 immunohistochemistry and their biological significance in colorectal cancer. J. Pathol..

[45] Norberg T., Lennerstrand J., Inganas M., Bergh J. (1998). Comparison between p53 protein measurements using the luminometric immunoassay and immunohistochemistry with detection of p53 gene mutations using cDNA sequencing in human breast tumors. Int. J. Cancer.

[46] Proestling K., Hebar A., Pruckner N., Marton E., Vinatzer U., Schreiber M. (2012). The Pro Allele of the p53 Codon 72 Polymorphism Is Associated with Decreased Intratumoral Expression of BAX and p21, and Increased Breast Cancer Risk. PLoS ONE.

[47] Post S.M., Quintas-Cardama A., Pant V., Iwakuma T., Hamir A., Jackson J.G., Maccio D.R., Bond G.L., Johnson D.G., Levine A.J. (2010). A high-frequency regulatory polymorphism in the p53 pathway accelerates tumor development. Cancer Cell.

[48] Wasielewski M., Nagel J.H., Brekelmans C., Klijn J.G., van den Ouweland A., Meijers-Heijboer H., Schutte M. (2007). MDM2 SNP309 accelerates familial breast carcinogenesis independently of estrogen signaling. Breast Cancer Res. Treat..

[49] Yarden R.I., Friedman E., Metsuyanim S., Olender T., Ben-Asher E., Papa M.Z. (2007). MDM2 SNP309 accelerates breast and ovarian carcinogenesis in BRCA1 and BRCA2 carriers of Jewish-Ashkenazi descent. Breast Cancer Res. Treat..

[50] Boersma B.J., Howe T.M., Goodman J.E., Yfantis H.G., Lee D.H., Chanock S.J., Ambs S. (2006). Association of breast cancer outcome with status of p53 and MDM2 SNP309. J. Natl. Cancer Inst..

[51] Campbell I.G., Eccles D.M., Choong D.Y. (2006). No association of the MDM2 SNP309 polymorphism with risk of breast or ovarian cancer. Cancer Lett..

[52] Ma H., Hu Z., Zhai X., Wang S., Wang X., Qin J., Jin G., Liu J., Wang X., Wei Q. (2006). Polymorphisms in the MDM2 promoter and risk of breast cancer: A case-control analysis in a Chinese population. Cancer Lett..

[53] Millikan R.C., Heard K., Winkel S., Hill E.J., Massa B., Mayes L., Williams P., Holston R., Conway K., Edmiston S. (2006). No association between the MDM2 -309 T/G promoter polymorphism and breast cancer in African-Americans or Whites. Cancer Epidemiol. Biomark. Prev..

[54] Forslund A., Zeng Z., Qin L.X., Rosenberg S., Ndubuisi M., Pincas H., Gerald W., Notterman D.A., Barany F., Paty P.B. (2008). MDM2 gene amplification is correlated to tumor progression but not to the presence of SNP309 or TP53 mutational status in primary colorectal cancers. Mol. Cancer Res..

[55] Deben C., Op de Beeck K., Van den Bossche J., Jacobs J., Lardon F., Wouters A., Peeters M., Van Camp G., Rolfo C., Deschoolmeester V. (2017). MDM2 SNP309 and SNP285 Act as Negative Prognostic Markers for Non-small Cell Lung Cancer Adenocarcinoma Patients. J. Cancer.

[56] Sanchez-Carbayo M., Socci N.D., Kirchoff T., Erill N., Offit K., Bochner B.H., Cordon-Cardo C. (2007). A polymorphism in HDM2 (SNP309) associates with early onset in superficial tumors, TP53 mutations, and poor outcome in invasive bladder cancer. Clin. Cancer Res..

[57] Alazzouzi H., Suriano G., Guerra A., Plaja A., Espin E., Armengol M., Alhopuro P., Velho S., Shinomura Y., Gonzalez-Aguilera J.J. (2007). Tumour selection advantage of non-dominant negative P53 mutations in homozygotic MDM2-SNP309 colorectal cancer cells. J. Med. Genet..

[58] Hori Y., Miyabe K., Yoshida M., Nakazawa T., Hayashi K., Naitoh I., Shimizu S., Kondo H., Nishi Y., Umemura S. (2015). Impact of TP53 Codon 72 and MDM2 SNP 309 Polymorphisms in Pancreatic Ductal Adenocarcinoma. PLoS ONE.

[59] Huang S.F., Chen I.H., Liao C.T., Wang H.M., Liou S.H., Hsieh L.L. (2009). Combined effects of MDM2 SNP 309 and p53 mutation on oral squamous cell carcinomas associated with areca quid chewing. Oral Oncol..

[60] Menin C., Scaini M.C., De Salvo G.L., Biscuola M., Quaggio M., Esposito G., Belluco C., Montagna M., Agata S., D’Andrea E. (2006). Association between MDM2-SNP309 and age at colorectal cancer diagnosis according to p53 mutation status. J. Natl. Cancer Inst..

[61] Langerod A., Bukholm I.R., Bregard A., Lonning P.E., Andersen T.I., Rognum T.O., Meling G.I., Lothe R.A., Borresen-Dale A.L. (2002). The TP53 codon 72 polymorphism may affect the function of TP53 mutations in breast carcinomas but not in colorectal carcinomas. Cancer Epidemiol. Biomark. Prev..

[62] Kaderi M.A., Mansouri M., Zainuddin N., Cahill N., Gunnarsson R., Jansson M., Kimby E., Aleskog A., Lundin J., Glimelius B. (2010). Lack of association between the MDM2 promoter polymorphism SNP309 and clinical outcome in chronic lymphocytic leukemia. Leuk. Res..

[63] Schmidt M.K., Tommiska J., Broeks A., van Leeuwen F.E., Van’t Veer L.J., Pharoah P.D., Easton D.F., Shah M., Humphreys M., Dork T. (2009). Combined effects of single nucleotide polymorphisms TP53 R72P and MDM2 SNP309, and p53 expression on survival of breast cancer patients. Breast Cancer Res..

[64] Toyama T., Zhang Z., Nishio M., Hamaguchi M., Kondo N., Iwase H., Iwata H., Takahashi S., Yamashita H., Fujii Y. (2007). Association of TP53 codon 72 polymorphism and the outcome of adjuvant therapy in breast cancer patients. Breast Cancer Res..

[65] Wang S., Chen L., Zhao Q., Rong H., Wang M., Gong W., Zhou J., Wu D., Zhang Z. (2013). Effect of TP53 codon 72 and MDM2 SNP309 polymorphisms on survival of gastric cancer among patients who receiving 5-fluorouracil-based postoperative adjuvant chemotherapy. Cancer Chemother. Pharm..

[66] De Martino M., Taus C., Wessely I.S., Lucca I., Hofbauer S.L., Haitel A., Shariat S.F., Klatte T. (2015). The T309G Murine Double Minute 2 Gene Polymorphism Is an Independent Prognostic Factor for Patients with Renal Cell Carcinoma. DNA Cell Biol..

[67] Kong Q., Li P., Tian Q., Ha M.W. (2014). Role of MDM2 T309G polymorphism in susceptibility and prognosis of nonsmall cell lung cancer: A meta-analysis. Genet. Test. Mol. Biomark..

[68] Nechushtan H., Hamburger T., Mendelson S., Kadouri L., Sharon N., Pikarsky E., Peretz T. (2009). Effects of the single nucleotide polymorphism at MDM2 309 on breast cancer patients with/without BRCA1/2 mutations. BMC Cancer.

[69] Taubenschuß E., Marton E., Mogg M., Frech B., Ehart L., Muin D., Schreiber M. (2013). The L10P Polymorphism and Serum Levels of Transforming Growth Factor β1 in Human Breast Cancer. Int. J. Mol. Sci..

[70] R Development Core Team (2009). R: A Language and Environment for Statistical Computing.

[71] Bender R., Lange S. (2001). Adjusting for multiple testing--when and how?. J. Clin. Epidemiol..

[72] Friesenhengst A., Pribitzer-Winner T., Miedl H., Pröstling K., Schreiber M. (2018). Elevated Aromatase (CYP19A1) Expression Is Associated with a Poor Survival of Patients with Estrogen Receptor Positive Breast Cancer. Horm. Cancer.

[73] Harrington D.P., Fleming T.R. (1982). A Class of Rank Test Procedures for Censored Survival-Data. Biometrika.

