# Supplementary material for: Association of the MDM2 SNP285 and SNP309 Genetic Variants with the Risk, Age at Onset and Prognosis of Breast Cancer in Central European Women: A Hospital-Based Case-Control Study
Source: Int J Mol Sci. 2019 Jan 25;20(3):509. doi: 10.3390/ijms20030509 (PMC6387136; doi:10.3390/ijms20030509)
Supplement: Supplementary file 1 [file ijms-20-00509-s001.pdf]

Table S1. Clinical characteristics and genotype frequencies of SNP309 in the study population.

			SNP309 genotype		
		**Total**	**TT**	**TG**	**GG**
All subjects		660	274 (41,5%)	300 (45,5%)	86 (13,0%)
Patients		406	169 (41,6%)	178 (43,8%)	59 (14,5%)
Controls		254	105 (41,3%)	122 (48,0%)	27 (10,6%)
Age (years)	<55	171	76 (44,4%)	74 (43,3%)	21 (12,3%)
	≥55	235	93 (39,6%)	104 (44,3%)	38 (16,2%)
Menopausal status	pre	100	46 (46,0%)	40 (40,0%)	14 (14,0%)
	post	250	100 (40,0%)	111 (44,4%)	39 (15,6%)
	na	56	23 (41,1%)	27 (48,2%)	6 (10,7%)
p53 status	pos	92	47 (51,1%)	38 (41,3%)	7 (7,6%)
	neg	259	104 (40,2%)	109 (42,1%)	46 (17,8%)
	na	55	18 (32,7%)	31 (56,4%)	6 (10,9%)
ER status	pos	265	117 (44,2%)	111 (41,9%)	37 (14,0%)
	neg	124	50 (40,3%)	54 (43,5%)	20 (16,1%)
	na	17	2 (11,8%)	13 (76,5%)	2 (11,8%)
PR status	pos	185	81 (43,8%)	76 (41,1%)	28 (15,1%)
	neg	198	82 (41,4%)	87 (43,9%)	29 (14,6%)
	na	23	6 (26,1%)	15 (65,2%)	2 (8,7%)
HER2 status	pos	74	32 (43,2%)	31 (41,9%)	11 (14,9%)
	neg	296	125 (42,2%)	129 (43,6%)	42 (14,2%)
	na	36	12 (33,3%)	18 (50,0%)	6 (16,7%)
KI67 pos cells	≤10%	183	82 (44,8%)	74 (40,4%)	27 (14,8%)
	>10%	125	49 (39,2%)	53 (42,4%)	23 (18,4%)
	na	98	38 (38,8%)	51 (52,0%)	9 (9,2%)
Stage	0, I	143	59 (41,3%)	68 (47,6%)	16 (11,2%)
	II-IV	186	83 (44,6%)	73 (39,2%)	30 (16,1%)
	na	77	27 (35,1%)	37 (48,1%)	13 (16,9%)
Grade	pG1-2	237	96 (40,5%)	104 (43,9%)	37 (15,6%)
	pG3	146	65 (44,5%)	61 (41,8%)	20 (13,7%)
	na	23	8 (34,8%)	13 (56,5%)	2 (8,7%)
Tumor size	pT1	175	81 (46,3%)	73 (41,7%)	21 (12,0%)
	pT2-4	158	64 (40,5%)	68 (43,0%)	26 (16,5%)
	other, na	73	24 (32,9%)	37 (50,7%)	12 (16,4%)
Tumor type	ductal	248	107 (43,1%)	108 (43,5%)	33 (13,3%)
	lobular	80	35 (43,8%)	32 (40,0%)	13 (16,3%)
	other, na	78	27 (34,6%)	38 (48,7%)	13 (16,7%)
Lymph node status	pN0	195	86 (44,1%)	87 (44,6%)	22 (11,3%)
	pN+	132	57 (43,2%)	55 (41,7%)	20 (15,2%)
	na	79	26 (32,9%)	36 (45,6%)	17 (21,5%)

Numbers of all patients and controls, as well as number of patients in the indicated subgroups are shown. Parenthesized numbers show the fraction of patients (%) with the indicated genotypes. ER, estrogen receptor; PR, progesterone receptor; na, status not available.

Table S2. Clinical characteristics and genotype frequencies of SNP285 in the study population.

			SNP285 genotype			
		Total	GG		GC	
All subjects		660	625	(94,7%)	35	(5,3%)
Patients		406	383	(94,3%)	23	(5,7%)
Controls		254	242	(95,3%)	12	(4,7%)
Age (years)	<55	171	160	(93,6%)	11	(6,4%)
	≥55	235	223	(94,9%)	12	(5,1%)
Menopausal status	pre	100	93	(93,0%)	7	(7,0%)
	post	250	239	(95,6%)	11	(4,4%)
	na	56	51	(91,1%)	5	(8,9%)
p53 status	pos	92	89	(96,7%)	3	(3,3%)
	neg	259	241	(93,1%)	18	(6,9%)
	na	55	53	(96,4%)	2	(3,6%)
ER status	pos	265	250	(94,3%)	15	(5,7%)
	neg	124	116	(93,5%)	8	(6,5%)
	na	17	17	(100,0%)	0	(0,0%)
PR status	pos	185	173	(93,5%)	12	(6,5%)
	neg	198	187	(94,4%)	11	(5,6%)
	na	23	23	(100,0%)	0	(0,0%)
HER2 status	pos	74	70	(94,6%)	4	(5,4%)
	neg	296	278	(93,9%)	18	(6,1%)
	na	36	35	(97,2%)	1	(2,8%)
KI67 pos cells	≤10%	183	176	(96,2%)	7	(3,8%)
	>10%	125	113	(90,4%)	12	(9,6%)
	na	98	94	(95,9%)	4	(4,1%)
Stage	0, I	143	137	(95,8%)	6	(4,2%)
	II-IV	186	175	(94,1%)	11	(5,9%)
	na	77	71	(92,2%)	6	(7,8%)
Grade	pG1-2	237	222	(93,7%)	15	(6,3%)
	pG3	146	138	(94,5%)	8	(5,5%)
	na	23	23	(100,0%)	0	(0,0%)
Tumor size	pT1	175	166	(94,9%)	9	(5,1%)
	pT2-4	158	148	(93,7%)	10	(6,3%)
	other, na	73	69	(94,5%)	4	(5,5%)
Tumor type	ductal	248	235	(94,8%)	13	(5,2%)
	lobular	80	77	(96,3%)	3	(3,8%)
	other, na	78	71	(91,0%)	7	(9,0%)
Lymph node status	pN0	195	185	(94,9%)	10	(5,1%)
	pN+	132	124	(93,9%)	8	(6,1%)
	na	79	74	(93,7%)	5	(6,3%)

Numbers of all patients and controls, as well as number of patients in the indicated subgroups are shown. Parenthesized numbers show the fraction of patients (%) with the indicated genotypes. The column "SNP285 CC" was omitted since none of the subjects exhibited this genotype. ER, estrogen receptor; PR, progesterone receptor; na, status not available.

Table S3. Association of *MDM2* SNP309 with breast cancer risk in the dominant inheritance model.

	Patients		SNP309		
	**Subgr.**	**Number (%)**	**OR**	**95% CI**	***p***
Age (years) [1]	<55	171 (42.1%)	0.88	0.60-1.30	0,526
	≥55	235 (57.9%)	1.08	0.75-1.54	0,691
Menopausal	pre	100 (28.6%)	0.83	0.52-1.32	0,425
status	post	250 (71.4%)	1.06	0.74-1.51	0,760
p53 status	pos	92 (26.2%)	0.67	0.42-1.09	0,107
	neg	259 (73.8%)	1.05	0.74-1.49	0,785
ER status	pos	265 (68.1%)	0.89	0.63-1.26	0.517
	neg	124 (31.9%)	1.04	0.67-1.61	0.850
PR status	pos	185 (48.3%)	0.90	0.62-1.33	0.609
	neg	198 (51.7%)	1.00	0.68-1.45	0.987
HER2 status	pos	74 (20.0%)	0.92	0.55-1.56	0.770
	neg	296 (80.0%)	0.96	0.69-1.35	0.833
Ki67 status	≤10%	183 (59.4%)	0.87	0.59-1.27	0.470
	>10%	125 (40.6%)	1.09	0.71-1.69	0.690
Stage	0 or I	143 (43.5%)	1.00	0.66-1.52	0.988
	II–IV	186 (56.5%)	0.87	0.60-1.28	0.492
Grade	pG1–2	237 (61.9%)	1.04	0.72-1.48	0.851
	pG3	146 (38.1%)	0.88	0.58-1.32	0.536
Tumor Size	pT1	175 (52.6%)	0.82	0.55-1.21	0.310
	pT2–4	158 (47.4%)	1.04	0.69-1.55	0.867

SNP309, GG + TG vs. TT (dominant model); Subgr., subgroup of patients; OR, odds ratios; 95% CI, 95% confidence intervals; *p*, *p*-values; pre, pre-menopausal; post, post-menopausal; ER, estrogen receptor; PR, progesterone receptor; pos, positive; and neg, negative; [1] patients aged under 55 years or ≥55 years at diagnosis were compared to control subjects of any age.

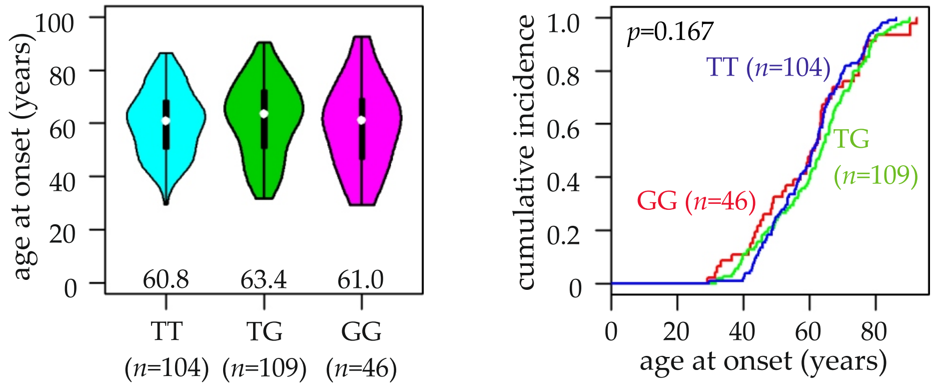

(a) SNP309 in *TP*53 negative patients

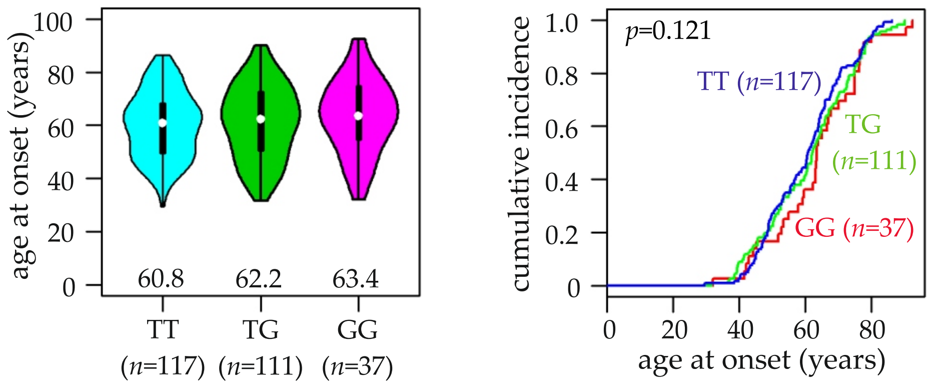

(b) SNP309 in ER positive patients

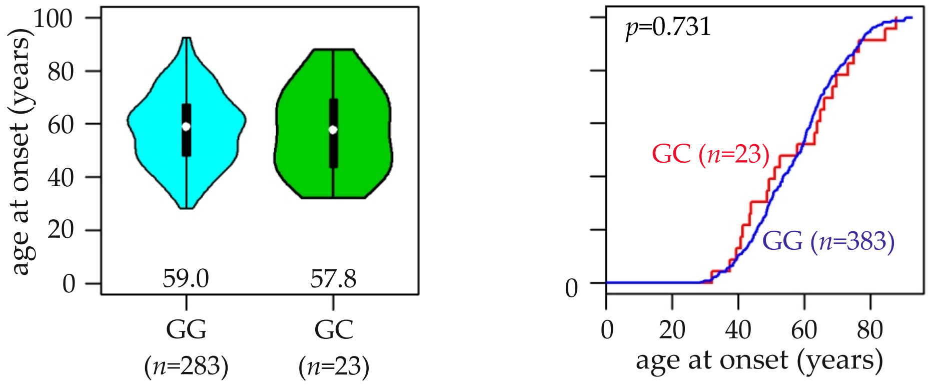

(c) SNP285 in all patients

Figure S1. Association of SNP309 and SNP285 genotypes with the age at breast cancer onset. Violin plots (left) and curves of the cumulative breast cancer incidence (right) of the indicated age at onset are shown for (**a**) SNP309 in *TP*53 negative patients, (**b**) SNP309 in estrogen receptor (ER) positive patients, and (c) SNP285 in unselected patients . Genotypes and numbers of patients (*n*) are indicated. Numbers in left panels indicate the median age at breast cancer onset of each genotype (indicated by white dots).
